# A Facile Chemical Synthesis of PbTe Nanostructures at Room Temperature

**DOI:** 10.3390/nano10101915

**Published:** 2020-09-25

**Authors:** Anil B. Gite, Balasaheb M. Palve, Vishwasrao B. Gaikwad, Gotan H. Jain, Habib M. Pathan, Samir Haj Bloukh, Zehra Edis

**Affiliations:** 1 Advance Physics Laboratory, Department of Physics, Savitribai Phule Pune University, Pune 411 007, India; gite.anil@gmail.com (A.B.G.); arjunpalve@gmail.com (B.M.P.); pathan@physics.unipune.ac.in (H.M.P.); 2 SNJB’sArts, Commerce Science College, Chandwad, Nashik 423 101, India; 3 Department of Chemistry, KTHM College, Nashik 422 002, Maharashtra, India; dr.gaikwadvb@rediffmail.com; 4 Department of Clinical Sciences, College of Pharmacy and Health Science, Ajman University, Ajman PO Box 346, UAE; s.bloukh@ajman.ac.ae; 5 Department of Pharmaceutical Sciences, College of Pharmacy and Health Science, Ajman University, Ajman PO Box 346, UAE

**Keywords:** lead telluride, PbTe, thermoelectric materials, chemical synthesis, nanostructures, cyclic voltammetry, electrical conductivity

## Abstract

Thermoelectric (TE) materials are possible solutions of the current problems in the energy sector to overcome environmental pollution, increasing energy demand and the decline of natural resources. Thermoelectric materials are a promising alternative for the conversion of waste heat to electricity. Nanocrystalline PbTe powder was synthesized by a simple chemical method at room temperature and systematically investigated at various durations as samples A1–A5. Fourier Transform infrared spectroscopy (FTIR), x-ray diffraction (XRD), microstructural analysis by scanning electron microscopy (SEM), and energy dispersive spectroscopy (EDS) confirmed the composition of the samples. TE parameters as thermo-emf of samples A1–A5 and electrical conductivity were measured. The cyclic voltammetry gives a band gap of 0.25 eV, which is in agreement with the optical band gap of the material. The A4 sample has an average crystal size of 36 nm with preferred orientation in (200) verifying the cubic morphology. The obtained TE parameters are beneficial for the non-uniform TE materials which might be due to strong current boundary scattering and extremely low thermal conductivity of the samples.

## 1. Introduction

Thermoelectric materials can convert heat to electricity [1]. These materials are environment friendly and mostly used for the development of sustainable energy materials. It has been reported that the narrow band material like PbTe has shown superior thermoelectric (TE) properties and is currently used in different applications [2,3]. These materials have prospective uses in thermal power generation and thermal sensing equipments. Diverse theoretical calculations and experimental data point to the upgrading in TE properties, which can be achieved by reducing the dimensionality of TE materials to a certain lowest amount level [2,4,5,6,7,8]. The dimensionless parameter figure of merit, which determines the efficiency TE material is ZT = (S^2^σT)/K, where S is Seebeck coefficient, σ is electrical conductivity, T is working temperature in Kelvin, and K is thermal conductivity of TE material [9]. A good TE material simultaneously demands large Seebeck coefficient (S), high electrical conductivity (σ), and low thermal conductivity (K). The thermal conductivity is due to the electron motion (Ke) and lattice vibration produced within the material (K_l_). This complex relationship between these TE parameters makes it difficult to enhance the ZT value of the TE materials. For that reason many efforts are aimed to improve the ZT value by balancing these interdependent TE parameters. Reducing the thermal conductivity via nanostructuring plays an important role for the improvement of the ZT of the material. For high ZT, high Seebeck coefficient is needed, but due to the complicated electronic band structures, superior electrical conductivity from high-symmetry cubic crystal structures, and low thermal conductivity caused by strong anharmonicity in TE materials, also due to the Pb local off-center, plays an important role in variations of TE parameters [10,11,12].

The quantum confinements will produce significant effects on charge and phonon transports in thermoelectric materials. Quantum confinement deals special way to manipulate carrier transports due to close connection between electronic band structure and dimensionality of the materials [13]. The Seebeck coefficient increased because of larger effective mass due to the band structure. PbTe material, indicating that reducing dimensionality of a given material is a potential possibility to enhance thermoelectric performance [14]. Studies of the film show a great interest in high performance and low dimensional TE materials since these materials perform better as compared to bulk materials [15]. PbTe is a great material for optoelectronic and in mid-infra-red ranges, also widely used in a large number of various TE devices [16]. The PbTe material also useful for the energy harvesting power generation [17,18]. Many thermoelectric materials are being explored for power generation applications, such as GeTe [19], PbTe [20,21], half-Heusler [22,23], and skutterudites [24,25]. Nanostructured materials that appear to be the most promising from a commercial point of view by virtue of their excellent thermoelectric performance and their high efficiency. The study thermoelectric nanomaterials are explored due to various approaches such as narrow bandgaps, heavy elements doping, point defects loading, and nanostructuring [26]. PbTe thin films were synthesized by various methods and have been utilized for research of PbTe material [27,28,29,30,31,32,33,34]. Wang et al. reported the simplistic chemical synthesis of PbTe on the glass substrate material at normal atmospheric conditions with temperatures around 300 K [35].

Chemical synthesis is one of the easiest methods for the synthesis of the material since it is a low-cost technique, which does not require any kind of sophisticated equipment, as well as specific kind of substrate materials. In the lead chalcogenide synthesis, PbX (PbX, X = Te, S, Se) material films are synthesized by chemical technique [36,37,38]. Seleno-sulfate (Na_2_SeSO_3_) is used widely as Se source by various groups for the synthesis of PbSe on glass substrate material [39,40,41]. Thiourea SC(NH₂)₂ or sodium thiosulfate (Na_2_S_2_O_3_xH_2_O) as sources of S are used for the synthesis of PbS on glass and silicon substrate materials [42,43,44].

Unfortunately, the corresponding Te source is difficult to obtain due to the scarce solubility of Te. Precursors like TeO_2_, Na_2_TeO_3_, and Na_2_TeSO_3_ are available, but unstable under natural conditions and therefore very rarely studies on chemical synthesis of PbTe are reported. In comparison to S or Se, the dis-proportionating reactions and hydrolysis of Te in different pH solutions are much more difficult [45]. Further problems are the cost effectiveness, a maximum ZT_max_, which is greater than 2.4 by the band convergence, and strain is produced in the lattice of PbTe [46]. Scarce and expensive material needed for the synthesis strain the opportunities to produce useful solutions for future energy problems. Recently, PbSe and PbS gained further attention due to their lower cost and higher operation temperature compared to Te [47,48].

However, the chemical synthesis of the PbTe material is easy and not frequently used due to the problem of hydrolysis of Te when compared to hydrothermal and solvothermal techniques, as well as solution phase synthesis and electro-deposition techniques [16,45,47,49,50,51,52,53,54,55].

In this study, we synthesized of PbTe powder by utilizing lead nitrate Pb(NO_3_)_2_ and tellurium oxide TeO_2_ as the precursors for Pb and Te from an alkaline aqueous solution bath. Lead telluride powder was prepared by a chemical method. High yield of PbTe for the various durations and formation mechanism of the PbTe powder and pellet was proposed and its TE properties of the film were measured for the better thermometric materials. We investigated in this work the synthesis of lead telluride powder by a chemical route technique. Silver-gray metallic powder of PbTe was successfully obtained at high yield. XRD reveals cubic nanocrystals of PbTe. The synthesized PbTe powder reveals homogeneous grains and agglomeration. The obtained values of the electrochemical band gap from the Cyclic Voltammetry are in agreement with the optical band gap. The formation of PbTe by chemical synthesis shall be a promising material that can be used as thermoelectric applications. Among the various synthesis techniques employed for the formation of PbTe nanostructures, chemical synthesis process has attracted much interest due to the advantage of high yield, low synthesis temperature, high purity, and high crystallinity.

## 2. Materials and Methods

### 2.1. Materials

Lead (II), telluride (TeO_2_, 99.998% trace metals basis), potassium hydroxide (KOH), trisodium-citrate (TSC) and potassium borohydride (KBH_4_) were purchased from Sigma Aldrich (St. Louis, MO, USA). Lead Nitrate (Pb(NO_3_)_2_, 99% pure) was obtained from Fisher Scientific (Pittsburgh, PA, USA). All the materials were pure and used as received. Additional purification was not done, when analytical grade precursors were utilized. Double distilled deionized water was used.

### 2.2. Preparation of Samples

**Figure 1:**
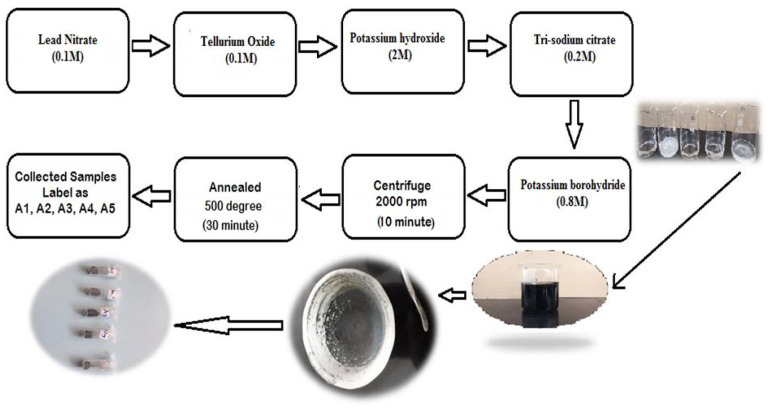
Step by step procedure for the synthesis of PbTe powder.

In a typical run, 0.1 M Pb(NO_3_)_2_, 0.1 M TeO_2_, 2 M KOH, 0.2 M tri-sodium citrate (TSC), and 0.8 M KBH_4_ were dissolved in sequence in 50 mL deionized water. Initially, a clean, colorless, transparent solution was formed. As time progressed, the color of the solution became dark gray and precipitation of dark gray material collected in the beaker for different durations as 72, 144, 216, 288, and 360 h. The solutions were then centrifuged for 10 min at 2000 rpm in a centrifuge machine. After centrifugation, the samples were collected in dry crucibles. Finally, the sample was dried in a vacuum at a temperature of 500 °C for 30 min. After the annealing, the collected grains were crushed to fine powder and the powder samples of different time durations were labeled as A1, A2, A3, A4, and A5. The samples were collected and analyzed for different characterization techniques and the TE properties of the samples were studied. Figure 1 represents the step-by-step procedure for the chemical bath deposition method used for the synthesis of PbTe.

### 2.3. Characterization of Samples

The samples A1–A5 were characterized by SEM/EDS, x-ray diffraction (XRD), FTIR, and cyclic voltammetry (CV). These methods confirmed the composition of our samples.

The PbTe structure was obtained by using the XRD–D8 Advance (Bruker, Karlsruhe, Germany) with Cu-Kα line wavelength 1.54 Å. The morphological study of the synthesized material under higher magnification was done by SEM from JEOL (JSM-6400, Tokyo, Japan) with an accelerating voltage of 20 kV of A1 and A5. The energy dispersive X-ray spectroscopy is used for the compositional analysis of the sample A1 and A5. For the FTIR analysis, JASCO FT/IR 6100 (Tokyo, Japan), with a range from 400 to 4000 cm^−1^ was used to find the presence of various modes in the material. Cyclic voltammetry (CV) measurements were carried out by K-Lyte 1.2 with the research applications of K-Lyte hardware (K-Lyte 1.2, from Knopy Techno Solutions, Kanpur, India). Seebeck coefficient measurements were done by a TEP unit TYPE-2, purchased from Borade Embedded Solutions, Kolhapur, India. Finally, for each sample, the pellets with dimensions 12 mm × 3 mm were prepared by a hydraulic press machine to find the thermo-emf of the synthesized material.

## 3. Results and Discussion

### 3.1. Characterization of Samples

**Figure 2:**
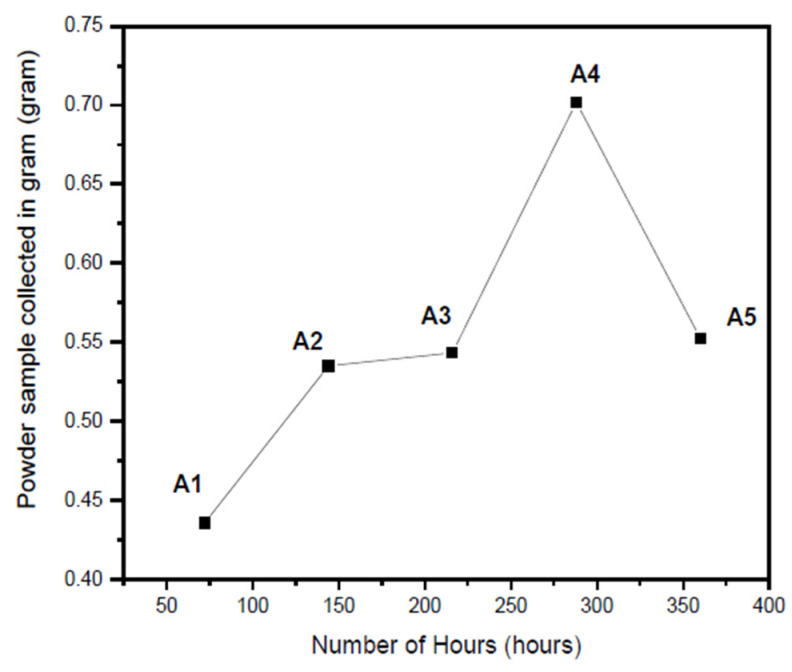
Yield of collected samples A1, A2, A3, A4, and A5 against the number of hours.

Qualitative analysis of the obtained yield was carried out by plotting the reaction time against the mass of the product (Figure 2).

Lead nitrate and tellurium oxide were dissolved in excess alkali and formed HPbO^2−^ and TeO_3_^2−^ ions during the reaction time. These ions further precipitate at the bottom. As time progressed, the rate of reaction and formation of PbTe may have been enhanced. This can be evident from the maximum yield in the case of sample A4. However, a further increase in the reaction time may have led to the overgrowth and thus, resulted in the deteriorated yield in the case of sample A5.

### 3.2. Cyclic Voltammetry (CV) Studies

**Figure 3:**
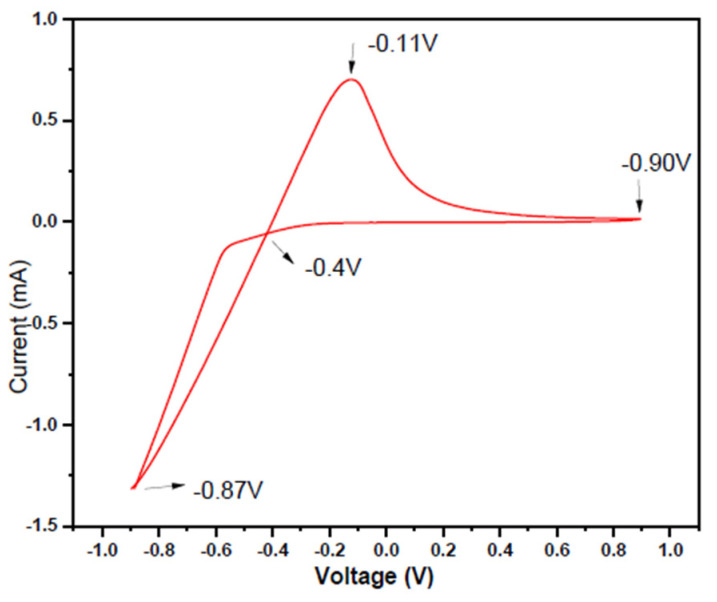
Cyclic Voltammogram of 0.1 M Pb(NO_3_)_2_ at a scan rate of 50 mV/s versus Pt tip working electrode and Ag/AgCl as a reference electrode.

CV studies revealed the appropriate potential ranges of the ions in the given electrolyte solutions. The curves give the scanning of the electrolyte in the cathodic direction and the negative current produced is called the cathodic currents (Figure 3).

**Figure 4:**
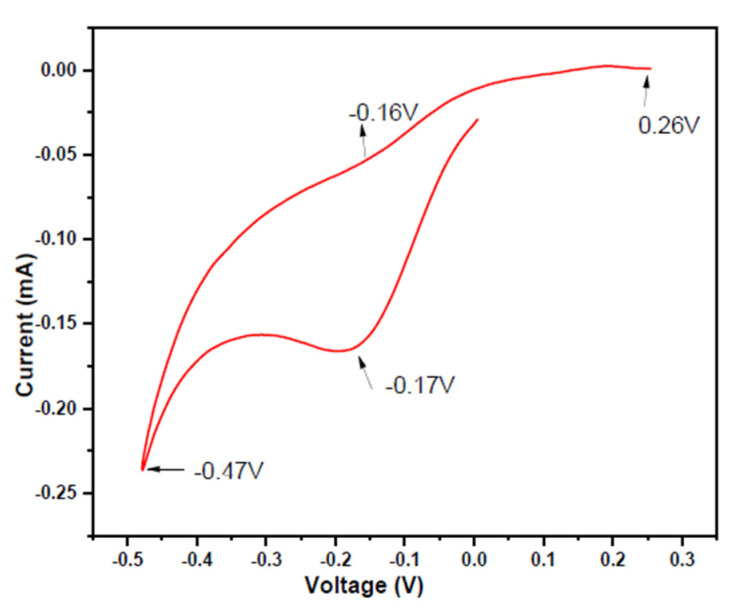
Cyclic Voltammogram of 0.1 mM HTeO^2+^ at scan rate of 50 mV/s versus Pt tip working electrode and Ag/AgCl as a reference electrode.

Figure 3 shows the deposition on the working electrode at a potential around −0.87 V versus Ag/AgCl, in the cathodic scan. The potential becomes more negative to the anodic scan and a strong oxidation peak was observed at −0.11 V. The precursor solution of lead nitrate in water as:

Pb(NO_3_)_2(s)_→ Pb_aq_^2+^ + 2 NO_3_^−^_aq_, (1)

with Pb_aq_^2+^ ion has standard reduction potential (E^0^) of—0.125 V and NO_3_^−^_aq_ ion in acidic conditions has E^0^ of +0.956 V [43,56]. The hysteresis was observed between the potential −0.87 V to +0.90 V indicating that reduction of Pb_aq_^2+^ occurs more rapidly on the Pt tip working electrode. The potential was negatively shifted versus Ag/AgCl and the reduction peak revealed the reduction of Te as shown in Figure 4.

**Figure 5:**
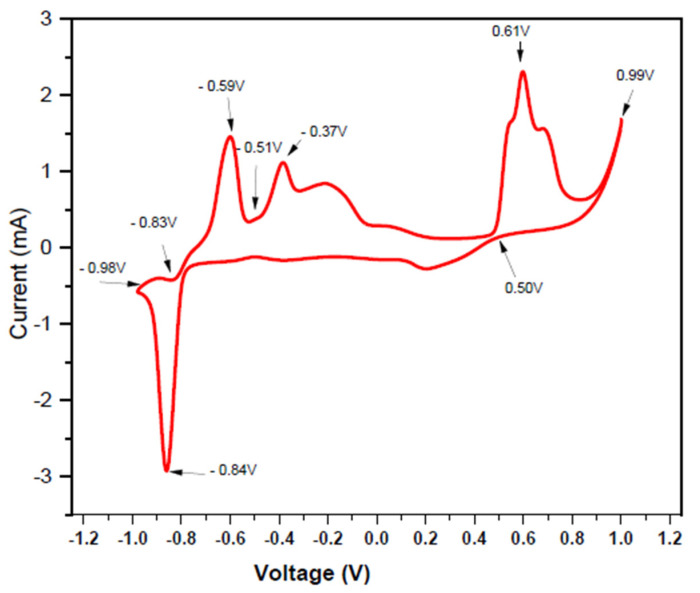
Cyclic Voltammetry of all the solutions when they are mixed together. Pt is used as working electrode, Pt mesh as a counter electrode and Ag/AgCl as reference electrode for sweep rate of 50 mV/s from −1.0 V to +1.0 V at the current limit of 1 mA by using K-Lyte 1.2 Potentiostat. Cyclic Voltagram of 0.1 M Pb(NO_3_)_2_, 0.1 M TeO_2_, 2 M KOH, 0.2 M trisodium citrate (TSC), and 0.8 M KBH_4_ were dissolved in sequence in 50 mL double distilled deionized water.

The reduction of Te can be represented as:

HTeO_2_^−^ + 3H^+^ + 4e^−^→ Te_ads_ + 2H_2_O, (2)

where Te_ads_ indicates that Tellurium atoms are absorbed in an electrolyte solution. When the potential reaches −0.17 V, another reduction wave started, which is attributed to the production of H_2_Te [41]. Each of the reduction peaks were some sort of limited deposition. This behavior involves the slow reduction of HTeO^2+^. Cyclic Voltammetry shows the presence of semiconductor material that has a discrete and fixed energy level. The correlation between the optical and electrochemical band gap was reported the first time by Haram et al. [56]. Figure 5 gives the electrochemical band gap and it is calculated from the peak values.

The obtained values are very well in agreement with an optical band gap:

ΔE = E_ox_ − E_red_ (eV), (3)

Values from the cyclic voltammetry curve E_ox_ = −0.59 V and E_red_ = −0.84 V give ΔE = 0.25 eV. The band gap of the PbTe is well matched with the observed band gap for the PbTe material.

### 3.3. Structural Analysis

**Figure 6:**
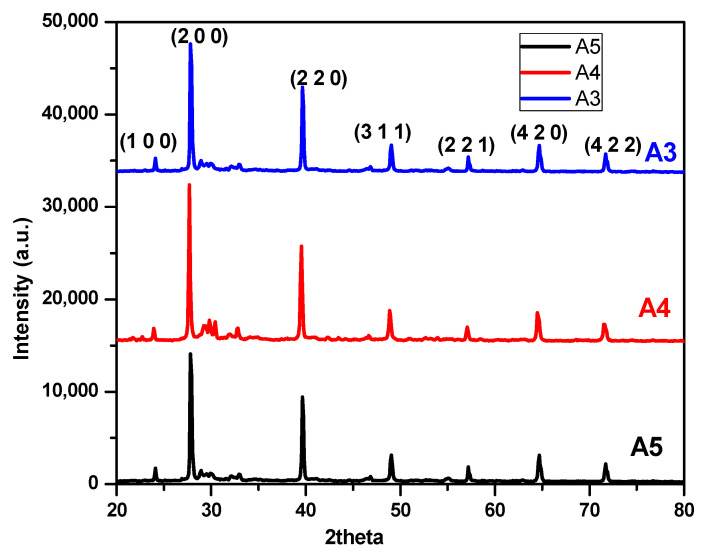
X-ray diffraction (XRD) analysis of samples A3, A4, and A5.

The average crystallite size was calculated from XRD data which is based on Debye Scherrer’s formula [57]:

D = 0.9λ/βcosθ,(4)

where D = average crystallite size, β = broadening of the diffraction line measured at half maximum intensity (FWHM), λ = wavelength of X-ray radiation, and θ = Bragg’s angle. The calculated d-spacing and crystallite sizes corresponding to various crystal planes are presented in Table 1. Figure 6 shows the typical XRD pattern for samples A3, A4, and A5 annealed after 500 °C.

All the diffraction signatures along the (h k l) plane can be attributed to the PbTe crystals. The powder samples A3, A4, and A5 give the well-matched peaks of their XRD patterns. The peak corresponding to (2 0 0) and (2 2 0) are the most prominent peaks as per the JCPDS file number 077-02460. Further strong intense peaks and other weak peaks are also indexed in the XRD pattern matching with the corresponding JCPDS file. The observed peaks were well matched with the XRD of undoped PbTe synthesized by solvothermal/hydrothermal process [58]. With the XRD of maximum yield sample A4 shows the formation of PbTe and reveals an average crystallite size of 36 nm for the A4 sample (Table 1).

The intensity and FWHM of XRD along (2 0 0) matches with the standard XRD pattern and thus, can be attributed to the prominent peak indicating the formation of the PbTe crystal in all the samples. This suggests that few crystals have followed the preferred orientation along (2 0 0). This may be evident from the independent small cubic morphologies shown in scanning electron micrographs. However, the XRD reveals additional weak orientations along (4 2 0) and (4 2 2) planes, which may have led to the agglomeration of the cubes in the bunch like morphologies. The dislocation density is defined as the length of dislocations lines per unit volume of crystal and calculated from the formula [59] as δ = 1/D^2^ where D is the crystal size and the micro-strain ε is given by ε = βcosθ/4. For the synthesized material the calculated values for dislocation density are 7.74 × 10^14^ and micro-strain produced in the synthesized material is 2.41 × 10^−3^.

### 3.4. Scanning Electron Microscope (SEM) and Elemental Studies

**Figure 7:**
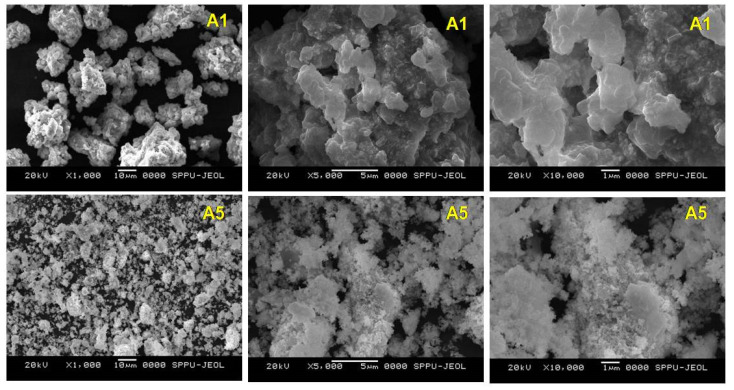
Scanning Electron Microscope (SEM) images of the sample at various magnifications of sample preparation.

Microstructural analysis by SEM/EDS was utilized for the morphological analysis of the samples, their sizes, and their composition. Figure 7 gives typical SEM images of sample A1 and A5 obtained at different magnifications.

**Figure 8:**
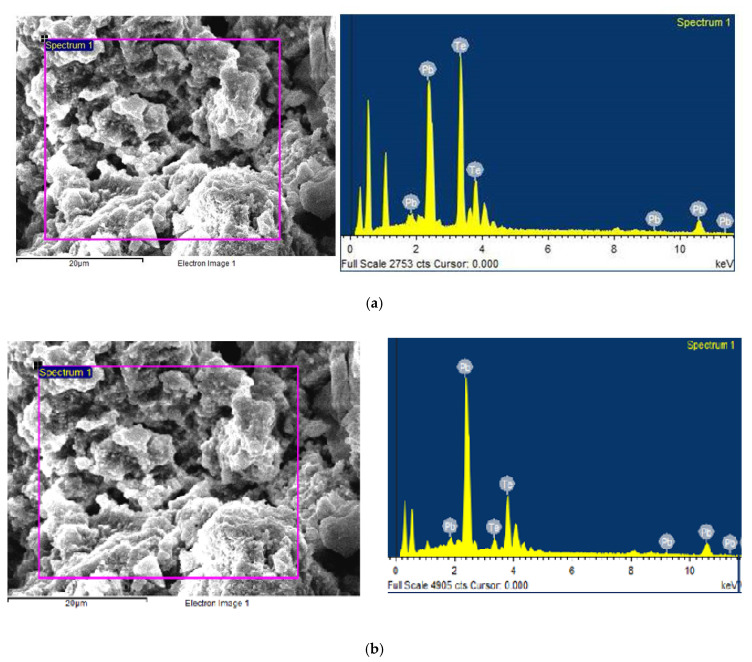
Energy dispersive spectroscopy (EDS) and corresponding scanning electron microscopy (SEM) of two samples. From up to down: (**a**) A1; (**b**) A5.

SEM reveals the formation of bunches of independent cubes. This may be attributed to the enhanced rate of reaction as a function of reaction time. Agglomerations of PbTe particles take place as the time progress as seen in the SEM images of sample A5. Elemental spectra of both the sample A1 and A5 reveal the strong peaks of Pb and Te as shown in Figure 8.

Besides, the observed additional peaks may be attributed to the physical adsorption of oxygen in the sample in air. Table 2 shows the composition of the A1 and A5 samples.

At shorter durations in the sample A1, the amount of Te was less than the amount of Pb with 45.43% and 54.57%, respectively. With increasing duration in sample A5, this changed to the opposite with increasing atomic percent of Te (53.15%) compared to Pb (46.85%), according to Table 2. The stoichiometric composition with atomic percentages should be in samples A1 and A5.

### 3.5. Fourier-Transform Infrared Spectroscopy (FTIR)

**Figure 9:**
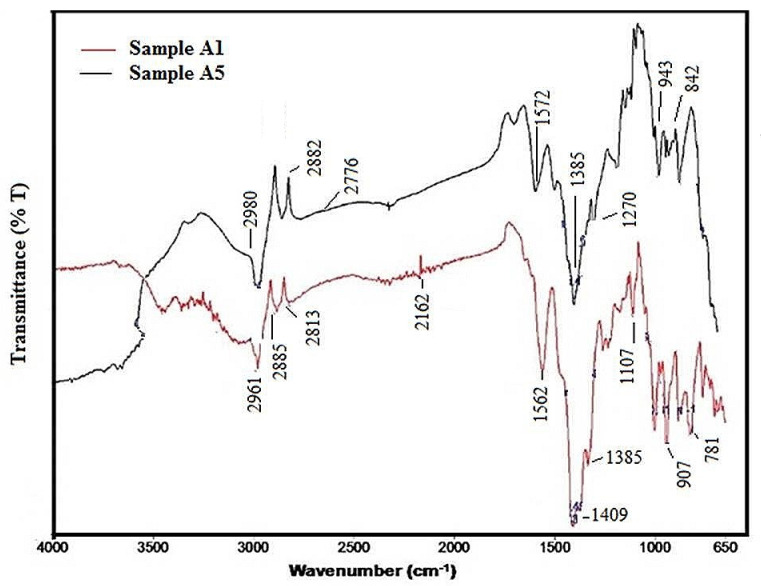
Fourier-transform-infrared (FTIR) spectrometric analysis of samples A1 and A5.

The optical characterization of prepared samples was performed with FTIR transmission (%T) measurements. FTIR analysis of the synthesized PbTe samples confirmed their composition and indicates the presence of various modes of PbTe in samples A1 and A5 (Figure 9). The organic or inorganic nature of the samples can be identified by using this technique for sample A1. The bands at 1409 cm^−1^, 1562 cm^−1^, 2885 cm^−1^, and 2813 cm^−1^ belong to the –CH_2_ outplane swinging, asymmetric (COO–) and symmetric –CH_2_ stretches in sample A1 (Figure 9). The bands at 2961 cm^−1^ and 2813 cm^−1^ are due to the –C–H stretching and anti-stretching vibrations of the –CH_2_ group, respectively, for the sample A1. This confirms the presence of stretching and bending modes of PbTe in the synthesized films. The bands around 1107 cm^−1^ to 1409 cm^−1^ might originate from –C–N stretching, –CH_2_ scissoring mode of vibration, and –OH bending mode. The bands between 781–907 cm^−1^ reveal vibrations of ternary amines with –CH_2_ rocking modes [54,60,61].

FTIR spectra of the sample A5 show that the strong absorption bands at 1572 cm^−1^, 1270 cm^−1^, and 1385 cm^−1^ are attributed to stretching vibrations of the carboxylate groups in the film. Various bands at 2980 cm^−1^, 2882 cm^−1^, and 2776 cm^−1^ are due to –CH stretching vibrations present in the structure of the PbTe [60]. The FTIR spectrum of the PbTe sample prepared by chemical precipitation at room temperature for 288 h of sample A5, indicates the presence of the bond of Pb–Te. The FTIR spectrum does not show strong bands associated with Pb–Se stretching, and bending vibrations, nor –CH_2_ Scissoring mode vibration, –C–N stretching, and –OH bending mode [61]. The vibrations of ternary amines are available at 842 to 943 cm^−1^ and the –CH_2_ rocking modes are observed between the 1270–1572 cm^−1^. FTIR transmittance bands are summarized in Table 3. The FTIR spectra of sample well matched with those reported earlier [54,60,61,62,63].

### 3.6. Thermo-Electromotive Force Measurements

**Figure 10:**
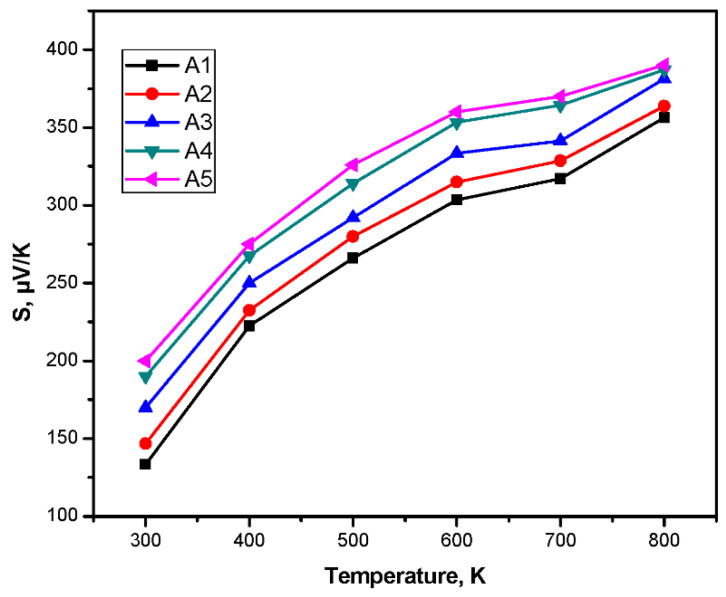
Plot of thermo-electromotive force versus temperature difference from two points on the pellets.

The thermo-electrical properties were studied in previous investigations [63]. The Seebeck coefficient of PbTe was measured by mounting the sample on two metal blocks, which enabled the generation of a temperature gradient, as reported in the previous studies [64]. For all measurements, one side contact was kept at room temperature (approximately 25 °C) with the help of circulating water at one side contact, while the other one was heated allowing the generation of a temperature gradient. A copper contact has been around the samples contact, in order to conduct the thermoelectrically driven voltage measurements. Silver paste was present between the copper contact and the sample surface. The copper contact from the two ends were fed to a multimeter-voltmeter and the generated thermo-voltage (V) was measured. The temperature gradient ΔT was measured. Then the Seebeck coefficient (S) was derived from the ratio of ΔV/ΔT. In the synthesis of the powder, pellets of the dimensions 12 (diameter) × 3 (height) mm were used in the measurements of the Seebeck coefficient (S) and electrical conductivity (σ) (Figure 10). Seebeck coefficient, as determined by laboratory made measurements system purchased from the Borade Embaded Solutions, provided the contacts mentioned above by using pellets formed from an exact size of the samples (A1–A5) respectively. The circular pellets of the dimensions were prepared by the hydraulic press machine, by application of high stress. Then, the thermo-emf of the samples was measured.

**Figure 11:**
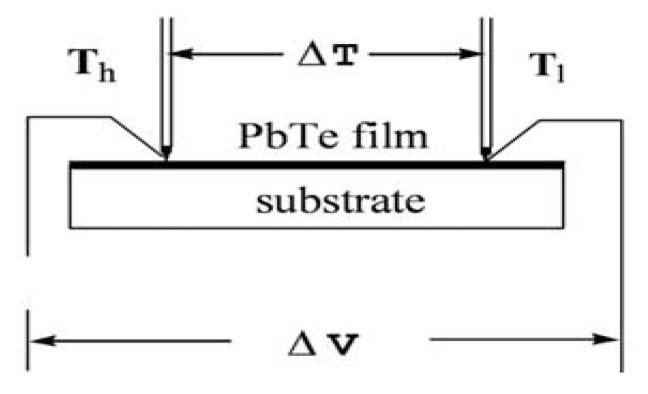
Schematic diagram of the Seebeck coefficient measurement.

Seebeck coefficient (S) for the samples A1 to A5 shows the p-type behavior with the variation of S from 50 µVK^−1^ to 380 µVK^−1^. The values of σ and S for the high yield sample were 23 Sm^−1^ and 314 µVK^−1^, respectively, and varied with temperature. Figure 10 shows the non-linear behavior of S with temperature T. These values are much in comparison to the gas evaporation method reported [65]. From the samples A1 to A5, there is increase in the thermo-emf as seen, since sample A5 shows the agglomeration at longer time durations in the synthesized sample. The sample A4 resulted in these values of σ and S due to strong grain boundary scattering of carriers, which may lead to extremely low thermal conductivity K for the synthesized material. This is more beneficial for the non-dimensional TE materials. With increase in thermo-emf, the electrical conductivity should also increase. The figure of merit, ZT (ZT = S^2^σT/k), determines the TE conversion efficiency of the TE material. The TE properties of the material could be increased by doping the other elements such as Se, Sn, Sb, and Ag. The decreased value of S is also observed when the PbTe is synthesized by the other methods and increase in the power factor of the material [66,67]. Figure 11 shows the schematic diagram of the Seebeck coefficient measurement.

As a result, the sample A4 is the best sample considering the yield produced and the measured values of the σ and S of the high yield sample. The synthesized PbTe pellet of high yield sample shows p-type conduction with electrical conductivity and Seebeck coefficient (S) as 23 Sm^−1^ and 314 µVK^−1^, respectively. This verifies that the higher quality of lead telluride material is formed at high yield. The plots of thermo-electromotive force versus temperature difference for all the samples from two points on the pellet film material indicate that the material shows the typical semiconducting of p-type behavior, and at higher temperature, the Seebeck coefficient of the samples almost remains the constant and decreasing nature. The sudden increase and decrease in the value of the thermo-emf indicates that the structural phase transition in the material will be observed during these temperature ranges of 500 K to 700 K (Figure 10).

## 4. Conclusions

Nanocrystalline PbTe powder was synthesized by a simple chemical method at room temperature and systematically investigated at various durations. This is a novel technique to synthesize the PbTe material which shows noble physico-chemical properties. Depending on the duration, samples were labelled as A1, A2, A3, A4, and A5. There is a direct relation between duration and yield of the sample until 288 h. After 288 h, the yield of sample A4 declined due to agglomeration. The cyclic voltammetry gives a band gap of 0.25 eV, which is very well in agreement with the optical band gap of the material. As compared to the other samples, the most prominent peaks of A4 sample in XRD pattern give the average crystal and size of 36 nm having preferred orientation in (2 0 0), verifying the cubic morphology. The dislocation density of the A4 sample is calculated as having a value of 7.74 × 10^14^ and the micro strain produced in the sample is 2.41 × 10^−3^. The lower yield of A1 and high yield of A5 sample surface is a result of the formation of independent cubes of PbTe having different magnifications in SEM studies. In the sample A5, as time progressed, the agglomeration of particles took place with the decrease in the yield. The various stretches produced in the lower yield A1 and maximum time duration A5 samples are observed in FTIR stretches, giving the presence of PbTe with various modes of vibrations in transmission spectra. The plots of thermo-electromotive force versus temperature difference for all the samples from two points on the films indicate that the film is a p-type conductor. The thermo-emf of all samples were measured to show the constant thermo-emf for all the samples, but higher yield sample A4 gives the well agreement values of electrical conductivity and thermo-emf as compared to the other samples. These values are beneficial for the non-uniform TE materials which might be due to strong current boundary scattering and extremely low thermal conductivity of the sample.

## Figures and Tables

**Table 1:** X-ray diffraction (XRD) analysis of the PbTe powder for sample A4.

(h k l)	2 (Degree)	d Spacing (Å)	FWHM (× 10^−3^)	Crystallite Size (nm)
(1 1 0)	24.15	3.72	2.004	42.37
(2 0 0)	27.94	3.22	2.004	42.70
(2 2 0)	39.91	2.28	4.004	22.04
(3 1 1)	49.10	1.86	2.004	45.55
(2 2 1)	57.30	1.61	4.008	23.61
(4 2 0)	64.60	1.44	2.004	49.02
(4 2 2)	72.04	1.31	4.008	25.62

**Table 2:** Elemental content of samples A1 and A5.

Sample	Element	Te (L)	Pb (M)	Total
A1	Weight %	7.82	15.25	23.06
	Atomic %	45.43	54.57	100
A5	Weight %	30.76	16.69	46.47
	Atomic %	53.15	46.85	100

**Table 3:** Fourier-transform-infrared (FTIR) spectrometric analysis of PbTe samples of sample A5.

Peak No.	X (cm^−1^)	Y (%T)	Peak No.	X (cm^−1^)	Y (%T)
1	2980.04	52.10	6	1270.56	58.49
2	2882.32	56.13	7	1152.32	62.51
3	2776.28	56.17	8	943.53	62.38
4	1572.67	58.11	9	908.60	63.56
5	1385.47	50.94	10	842.71	61.07
